# 

**Published:** 1886-11

**Authors:** 


					﻿N O V E M B E R , 1 8 8 6 .
OLD SERIES
Vol. XXV
JNDEXE.	|
xg. CM0AA SERIES
<*f®us4£s
1855 4

faGAl^JRW
P Journo

1 w.f.westmoreDand. ®■$
H.VM.M1LLER.M.D.LL .D.jBr
JAMES A.GRAY.
IH
MUSHED KY
JAMES P.HARRISON&CQS
♦Atlanta ,ga. Jm
SUBSCRIPTION: S2.5O A YEAR.
				

